# Sperm Flagellum Volume Determines Freezability in Red Deer Spermatozoa

**DOI:** 10.1371/journal.pone.0112382

**Published:** 2014-11-07

**Authors:** José Luis Ros-Santaella, Álvaro Efrén Domínguez-Rebolledo, José Julián Garde

**Affiliations:** 1 SaBio, Instituto de Investigación en Recursos Cinegéticos (IREC), CSIC-UCLM-JCCM, Campus Universitario, Albacete, Spain; 2 Department of Animal Science and Food Processing, Faculty of Tropical AgriSciences, Czech University of Life Sciences, Prague, Czech Republic; 3 Instituto Nacional de Investigaciones Forestales, Agrícolas y Pecuarias (INIFAP), Mocochá, Yucatán, México; Universidad Nacional Autónoma de México, Mexico

## Abstract

The factors affecting the inter-individual differences in sperm freezability is a major line of research in spermatology. Poor sperm freezability is mainly characterised by a low sperm velocity, which in turn is associated with low fertility rates in most animal species. Studies concerning the implications of sperm morphometry on freezability are quite limited, and most of them are based on sperm head size regardless of the structural parts of the flagellum, which provides sperm motility. Here, for the first time, we determined the volumes of the flagellum structures in fresh epididymal red deer spermatozoa using a stereological method under phase contrast microscopy. Sperm samples from thirty-three stags were frozen and classified as good freezers (GF) or bad freezers (BF) at two hours post-thawing using three sperm kinetic parameters which are strongly correlated with fertility in this species. Fourteen stags were clearly identified as GF, whereas nineteen were BF. No significant difference in sperm head size between the two groups was found. On the contrary, the GF exhibited a lower principal piece volume than the BF (6.13 µm^3^ vs 6.61 µm^3^, respectively, p = 0.006). The volume of the flagellum structures showed a strong negative relationship with post-thawing sperm velocity. For instance, the volume of the sperm principal piece was negatively correlated with sperm velocity at two hours post-thawing (r = −0.60; p<0.001). Our results clearly show that a higher volume of the sperm principal piece results in poor freezability, and highlights the key role of flagellum size in sperm cryopreservation success.

## Introduction

It is well known that the cryopreservation process negatively affects the viability and fertility of reproductive cells. There are several factors caused by cryopreservation protocols that alter sperm integrity, such as a change in the temperature of the diluents [1]; osmotic and toxic stresses induced by cryoprotectants [1], [2]; formation/reshaping of intracellular ice during freezing and thawing [3]; and dissolution of ice in the extracellular environment [2]. All of these factors induce sperm volumetric changes, plasma membrane alterations, flagellum morphological defects, as well as decrease mitochondrial membrane potential, sperm motility, viability, and fertility [2], [4]–[10]. As a generalisation, some 40–50% of the sperm population does not survive cryopreservation even with optimised protocols [2]. Thus, many of the frozen-thawed spermatozoa show a shorter life span and have difficulties in reaching the oocytes and penetrating their vestments after conventional artificial insemination [10]. Variations between individuals in sperm freezability have been reported in numerous animal species. Within this context, semen donors have routinely been categorized as “good freezers” (GF) or “bad freezers” (BF). When experiments involve comparing bulk samples before freezing and after thawing, it is difficult to know which parts of the cryopreservation procedure may be causing problems and to detect differences between individual cells [11]. In boar sperm, it has been demonstrated that these consistent inter-individual variations in sperm freezability are genetically determined [12].

The understanding and prediction of the sperm functional response to cryopreservation is one of the major questions in sperm cryobiology [13]. For this reason, sperm morphometry classification has become an integral part of routine sperm analysis. In recent years, ASMA (Automated Sperm Morphometry Analysis) systems have been employed for determining morphometric parameters of the sperm head and midpiece to elucidate possible relations with sperm freezability [3], [14]. These systems can only analyse the lengths and areas of the sperm head and midpiece, but they are unable, for example, to measure the sperm principal piece, which provides sperm motility. Thus, in order to study sperm midpiece morphometry with these systems, it is not easy to find a staining method which allows discerning this structure from the rest of the flagellum [15]. By contrast, with other software for analysis and image processing, it is possible to obtain the lengths of flagellum structures. Numerous studies argue the implications of sperm flagellum in several biological processes such as sperm velocity, male reproductive success, relationships with testicle size, and spermatogenesis [16]–[21]. On the other hand, studies concerning the role played by flagellum size on sperm freezability have not yet been reported, although there is evidence of the fragility of its internal and external structures when they are exposed to the cryopreservation process [5], [7], [22]–[24].

Curry et al. [25] reported that the sperm area and volume have great importance for determining optimal sperm cryopreservation protocols. For this purpose, stereology is able to accurately estimate surface areas and volumes for complex shapes from two-dimensional images [25]. Thereby, sperm volumes as well as the sperm head and flagellum structures can be examined. The sperm volumes of human and several mammalian species have been determined by stereological methods [19], [25].

In the present study, we hypothesized that flagellum volume could predict freezability in red deer spermatozoa. For this reason, we focused on determining the volumes of the sperm flagellum, midpiece, and principal piece in fresh sperm using stereology under phase contrast microscopy. In this way, differences between males in sperm flagellum volume could be linked to sperm freezability, owing to the fragility of flagellum structures to the cryopreservation process [5], [7], [23], and the differences observed among red deer sperm to support cryopreservation [14]. In order to determine sperm freezability, sperm samples were classified as good or bad freezers using three kinetic parameters (VAP: average path velocity, VCL: curvilinear velocity, and VSL: straight linear velocity) assessed by a CASA (Computer Assisted Sperm Analysis) system at 2 hours post-thawing and with incubation at 37°C. Sperm viability, acrosomal status, and mitochondrial activity were also determined by flow cytometry.

## Materials and Methods

### Animals

The study was approved by the “Comité de Ética en Investigación de la Universidad de Castilla-La Mancha”. All animal handling was done following the Spanish Animal Protection Regulation RD 53/2013, which conforms to the European Union Regulation 2003/65. Stags were legally culled and hunted in their natural habitat in accordance with the harvest plan of the game reserve. The harvest plans were made following the Spanish Harvest Regulation, Law 2/93 of Castilla-La Mancha, which conforms to European Union regulations. Thirty-three stags (age >4.5 years; body mass >130 kg) of red deer (*Cervus elaphus*) were used in this study. Landowners and managers of the red deer populations gave permission to the authors to use the samples.

### Stags and testes collection

Testes were recovered from adult red deer culled during the 2008 hunting season in the south of Spain. Testes, together with the scrotum, were removed and transported at approximately 20–22°C to the laboratory. The time that elapsed between animal death and sperm recovery ranged from 3 to 6 hours, an adequate and reliable time interval for evaluating sperm parameters, because a decrease in the quality of sperm traits begins to take place 12 hours after the death of a male [26].

### Chemicals and solutions

Unless otherwise stated, chemicals were obtained from Sigma-Aldrich (Madrid, Spain).

A Salamon's modified extender was prepared in two fractions, as previously described [27]. Fraction A contained: Tris (2.70%, w/v), fructose (1%, w/v), citric acid (1.4%, w/v), and clarified egg yolk (20%, v/v) (pH 6.8, osmolality 300 mOsm/Kg). Fraction B differed from the Fraction A in that water was replaced (12%, v/v) with the same volume of glycerol, with a final concentration  = 6% (v/v). Glutaraldehyde solution was composed of 2% glutaraldehyde (v/v), and 0.165 mol/L cacodylate/HCl buffer (pH 7.3), as previously described [27]. Bovine gamete medium (BGM-3) was composed of 87 mmol/L NaCl, 3.1 mmol/L KCl, 2 mmol/L CaCl_2_, 0.4 mmol/L MgCl_2_, 0.3 mmol/L NaH_2_PO_4_, 40 mmol/L HEPES, 21.6 mmol/L sodium lactate, 1 mmol/L sodium pyruvate, 50 µg/mL kanamicine, 10 µg/mL phenol red, and 6 mg/mL BSA (Bovine Serum Albumine) (pH 7.5), as previously described [28].

### Cryopreservation of epididymal spermatozoa

Post-mortem seminal recovery is the most practical option to obtain sperm samples from wild populations of red deer, with hunting representing a constant source from harvested animals [29]. Spermatozoa were collected from the cauda of the epididymis by repeated longitudinal and transverse cuts with a surgical scalpel and placed in 0.5 mL of fraction A. The contents from both epididymides of each individual were pooled for processing. Afterward, a routine sperm evaluation was made. Sperm concentration was determined using a Bürker counter chamber. The percentage of motile sperm and the quality of motility (QM) were subjectively evaluated, the latter using a scale from 0 (lowest: immobile) to 5 (highest: progressive and vigorous movement). Then, the sperm motility index (SMI) was calculated according to the formula [30]: 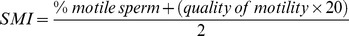



Only samples with at least 60% motile sperm were used for this study (Table S1). The sperm cryopreservation protocol was performed as previously described [27]. Briefly, the sperm mass was diluted at room temperature to 400×10^6^ spermatozoa/mL with fraction A, and then to 200×10^6^ spermatozoa/mL with fraction B. The diluted samples were refrigerated for approximately 10 min to reach 5°C and then equilibrated at the same temperature for 2 h. After equilibration, the suspended sperm was loaded into 0.25 mL plastic straws and frozen for 10 min in nitrogen vapours, 4 cm above the level of liquid nitrogen (−120°C). The straws were then immediately immersed into liquid nitrogen (−196°C) for storage.

### Morphometry assessment of fresh sperm

**Figure 1:**
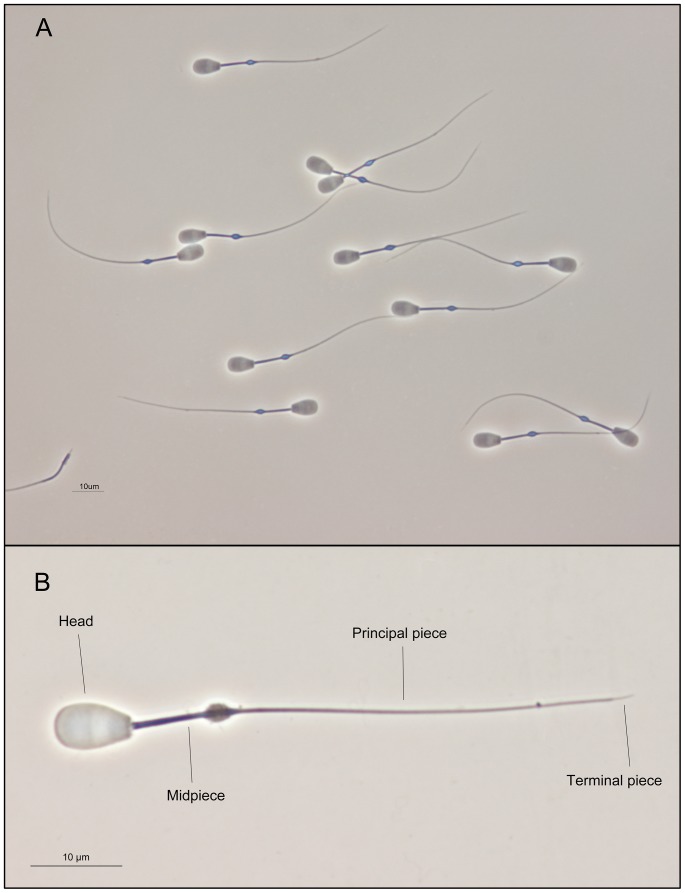
Red deer sperm. (A) It is remarkable how the method described in this study avoids floating cells. The picture was taken under phase contrast microscopy (40X objective). Scale bar, 10 µm. (B) Main structures of red deer spermatozoon. The picture was taken under phase contrast microscopy (100X objective) using the method described in the present study. Scale bar, 10 µm.

Sperm samples were directly recovered from the cauda of both epididymides. Spermatozoa were fixed in glutaraldehyde solution. A sub-sample of 2 µl was used to prepare the smears. Semen smears were air-dried for one day, then immersed in the glutaraldehyde fixative solution for 5 min and immediately mounted, sealing the edges with dibutyl phthalate xylene (DPX). This method avoids floating cells on the slide (Figure 1A), which greatly helps sperm morphometry analysis. Sperm samples were photographed using a high-resolution camera DXM1200 (Nikon, Tokyo, Japan) under a phase-contrast microscopy using an Eclipse E600 microscope (Nikon, Tokyo, Japan), and a 40X objective (Nikon, Tokyo, Japan). The resolution of the pictures was 3840×3072 pixels (TIFF format). A scale of 10 µm (181 pixels) was used for the measurements. The pixel size was 0.055 µm in the horizontal and vertical axes. Sperm lengths were assessed using ImageJ software (National Institutes of Health, USA). The main structures of red deer spermatozoon are shown in Figure 1B. The following sperm morphometry parameters were determined: head width, head length, proximal midpiece width, distal midpiece width, midpiece length, flagellum length, and terminal piece length (Table S1). From these measurements, we calculated other morphometric parameters such as total sperm and principal piece lengths. The head area was calculated using the formula for the area of an ellipse [31], [32]: 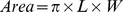



Head perimeter was calculated using the Ramanujan's formula for calculating the perimeter of an ellipse [33]:
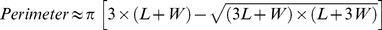



In both formulae, L and W are the semi-major and semi-minor axis of the sperm head, respectively. Twenty-five representative sperm were measured for each male as described by Malo et al. [16].

#### Stereology of the flagellum

Sperm flagellum volume and its structures were determined using a stereology method based on Anderson et al. [19] with some modifications. Anderson et al. [19] calculated the volumes of the sperm midpiece and flagellum using the formula for the volume of a cylinder. In this study, owing to the significant differences between the proximal and distal midpiece widths (0.94 µm and 0.74 µm, respectively; p<0.0001), we estimated sperm midpiece volume using the formula for the volume of a truncate cone:
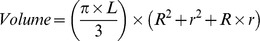
where L is the length of the midpiece, R is the half proximal midpiece width, and r is the half distal midpiece width. On the other hand, the total flagellum and principal piece volumes were determined using the formula for the volume of a cone:
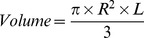
where R is the half midpiece width (i.e., proximal or distal) and L is the length of the flagellum or the principal plus terminal piece.

### Sperm thawing and sperm quality assessment

The sperm straws were thawed in a water bath with a saline solution at 37°C for 30 s, and each sample was poured in a tube. The samples were incubated at 37°C and analysed for motility (subjectively and with the CASA system), viability, acrosomal status, and mitochondrial status at 0 hours (after 10 min of incubation) and after 2 hours post-thawing (Table S2 and Table S3, respectively).

#### CASA analysis

Sperm were diluted down to 25–30×10^6^ spermatozoa/mL with fraction A solution and loaded into a Makler counter chamber (Sefi-Medical instruments, Haifa, Israel) at 37°C. The CASA system consisted of a triocular optical phase contrast microscope Eclipse 80i (Nikon, Tokyo, Japan), equipped with a warming stage at 37°C and a Basler A302fs digital camera (Basler Vision Technologies, Ahrensburg, Germany). Images were captured and analysed using the Sperm Class Analyzer software (Microptic S.L., Barcelona, Spain). The analysis was carried out using a 10X negative phase-contrast objective (Nikon, Tokyo, Japan). A total of 4 descriptors of sperm motility were recorded analysing a minimum of 250 sperm per sample: average path velocity (VAP, µm/s), curvilinear velocity (VCL, µm/s), straight linear velocity (VSL, µm/s), and amplitude of lateral head displacement (ALH, µm). The standard parameters settings were as follows: 25 frames/s; 20 to 60 µm^2^ for the head area.

#### Fluorescence probes for sperm viability, acrosomal status, and mitochondrial activity

Several physiological traits were assessed using fluorescent probes and flow cytometry, as previously described [28]. Briefly, the samples were diluted down to 10^6^ spermatozoa/mL in BGM-3 solution and stained using four fluorophores. Sperm viability was assessed with 0.1 µmol/L YO-PRO-1 (Invitrogen, Barcelona, Spain) and 10 µmol/L PI (propidium iodide). Mitochondrial activity and acrosomal status were assessed with 0.1 µmol/L Mitotracker Deep Red (Invitrogen, Barcelona, Spain) and 4 µg/mL PNA-TRITC (peanut agglutinin), respectively. The spermatozoa stained in these two solutions were incubated 20 min in the dark before being run through a flow cytometer. The sperm populations shown in this work were: YO-PRO-1-/PI- (viable spermatozoa), MT+ (Mitotracker Deep Red, spermatozoa with active mitochondria), and PNA- (spermatozoa with intact acrosome).

#### Flow cytometry analysis

Samples were analysed as previously described [28]. Briefly, a Cytomics FC500 flow cytometer (Beckman Coulter, Brea, CA, USA) was utilized, with a 488-nm Ar-Ion laser (excitation for YOPRO-1, PNA-TRITC, and PI), and a 633-nm He-Ne laser (excitation for Mitotracker Deep Red). The FSC (forward-scattered light) and SSC (side-scattered light) signals were used to gate out debris (non-sperm events). Fluorescence from YO-PRO-1 was read using a 525/25BP filter, PNA-TRITC was read using a 575/20BP filter, PI was read using a 615DSP filter, and Mitotracker Deep Red was read using a 675/40BP filter. Fluorescence captures were controlled using the RXP software provided with the cytometer. All of the parameters were read using logarithmic amplification. For each sample, 5000 spermatozoa were recorded, saving the data in flow cytometry standard (FCS) v. 2 files. The analysis of the flow cytometry data was carried out using WEASEL v. 2.6 (WEHI, Melbourne, Victoria, Australia).

### Statistical analysis

All statistical analyses were performed using the SPSS 20.0 statistical software package (SPSS Inc, Chicago, IL, USA). Sperm samples from thirty-three (N = 33) red deer were used for statistical analysis. The Kolmogorov-Smirnov test was used to check the normal distribution of the data. A repeated measures one-way ANOVA test was used to compare subjective motility between fresh and thawed sperm (0 and 2 hours) using the Mauchly's sphericity test (Greenhouse-Geisser correction) to verify homogeneity of variance. The quality of motility (QM) was not normally distributed in some groups, therefore, non-parametric Friedman test (repeated measures) was used. On the other hand, to check differences in sperm parameters (kinetics-CASA and flow cytometry) between 0 and 2 hours post-thaw we used a paired-samples t-student test (repeated measures). In order to determine sperm freezability we performed a hierarchical clustering analysis using the Euclidean distance measure after determining automatically the number of conglomerates by cluster analysis in two phases. For this purpose, we used three sperm kinetic parameters (VAP, VCL, and VSL) because they are highly related with fertility in red deer [34] and are good indicators of post-thawing sperm quality [35]. Sperm freezability was determined at 2 hours post-thawing to test spermatozoa thermo-resistance. The independent-samples student-t test (Levene's test to verify homogeneity of variance) and the Mann-Whitney U test were used to check for differences in sperm parameters between the GF and the BF.

On the other hand, since the variables of sperm velocity (VAP, VCL, VSL, ALH, and SMI) were highly correlated among themselves, we reduced the number of predictor variables using principal component analysis (PCA) to obtain an overall sperm velocity at 0 and 2 hours post-thawing, respectively. Bartlett sphericity and Keiser-Meyer-Olkin tests were assessed as measures of sampling adequacy [36]. Pearson's correlation test and the linear regression model were used to assess the relationship between sperm velocity and morphometric parameters. We used the RMA software to reduced major axis regression [37]. The within male coefficient of variation (CV) in sperm morphometry was calculated to show the intra-male variability in sperm design.

## Results

### Effects of sperm cryopreservation

After the freezing-thawing process, a remarkable decrease in subjective motility parameters was observed between fresh and thawed sperm, but also between 0 and 2 hours post-thawing: motile sperm, *F*(1.49, 47.55) = 94.27, p<0.001; QM, *χ* ^2^(2, N = 33) = 37.83, p<0.001; and SMI, *F*(1.51, 48.22) = 116.02, p<0.001 (Table 1). In the same way, there were significant differences for all of the CASA kinetic parameters between 0 and 2 hours post-thawing: VAP, t(32) = 9.77, p<0.001; VCL, t(32) = 10.46, p<0.001; VSL, t(32) = 10.29, p<0.001; and ALH, t(32) = 10.18, p<0.001 (Table 2). Furthermore, sperm viability and organelle functionality showed a decrease after thawing, displaying highly significant differences between 0 and 2 hours post-thawing: YOPRO-1-/PI-, t(32) = 11.47, p<0.001; MT+, t(32) = 6.26, p<0.001; and PNA-, t(32) = 11.18, p<0.001 (Table 2). For instance, the mean percentage of sperm with active mitochondria ranged from 51.13% to 28.63% during incubation (at 0 and 2 hours, respectively).

**Table 1:** Subjective motility of fresh and thawed red deer spermatozoa (N = 33).

	Sperm motility		
	Motile sperm (%)	QM (0–5)	SMI (%)
Fresh sperm	81.82±11.44^a^	2.24±0.48^a^	63.33±9.11^a^
0 hours post-thaw	55.91±16.32^b^	1.86±0.28^a^	46.51±9.96^b^
2 hours post-thaw	42.57±13.52^c^	1.43±0.21^b^	35.61±7.71^c^

Different superscript letters within the same column differ significantly (p<0.001). Data are shown as mean ± SD (standard deviation). QM, quality of motility; SMI, sperm motility index.

**Table 2:** Kinetics, viability, and organelle status of red deer spermatozoa (N = 33) at 0 and 2 hours post-thaw.

Assessed parameters	0 hours post-thaw		2 hours post-thaw	
	Mean ± SD	Range	Mean ± SD	Range
Sperm kinetics				
VAP (µm/s)	60.60±13.48^a^	26.08-87.21	40.09±10.41^b^	23.18–57.16
VCL (µm/s)	97.58±21.52^a^	55.49–144.36	66.60±14.14^b^	43.92–95.45
VSL (µm/s)	34.17±7.17^a^	15.56–49.03	22.17±5.00^b^	14.11–29.44
ALH (µm)	3.78±0.80^a^	2.49–5.47	2.69±0.43^b^	2.05–3.55
Sperm viability and organelle status				
Viability (YOPRO-1-/PI-, %)	35.81±8.91^a^	18.46–51.16	29.70±8.28^b^	13.20–41.32
Active mitochondria (MT+, %)	51.13±13.93^a^	26.42–78.51	28.63±19.00^b^	0.88–56.08
Intact acrosome (PNA-, %)	83.96±6.82^a^	67.40–93.96	69.67±11.16^b^	50.74–90.72

Different superscript letters within the same row differ significantly (p<0.001) between 0 and 2 hours post-thaw. SD, standard deviation; VAP, average path velocity; VCL, curvilinear velocity; VSL, straight linear velocity; ALH, amplitude of lateral head displacement.

### Deer sperm freezability (GF and BF)

**Figure 2:**
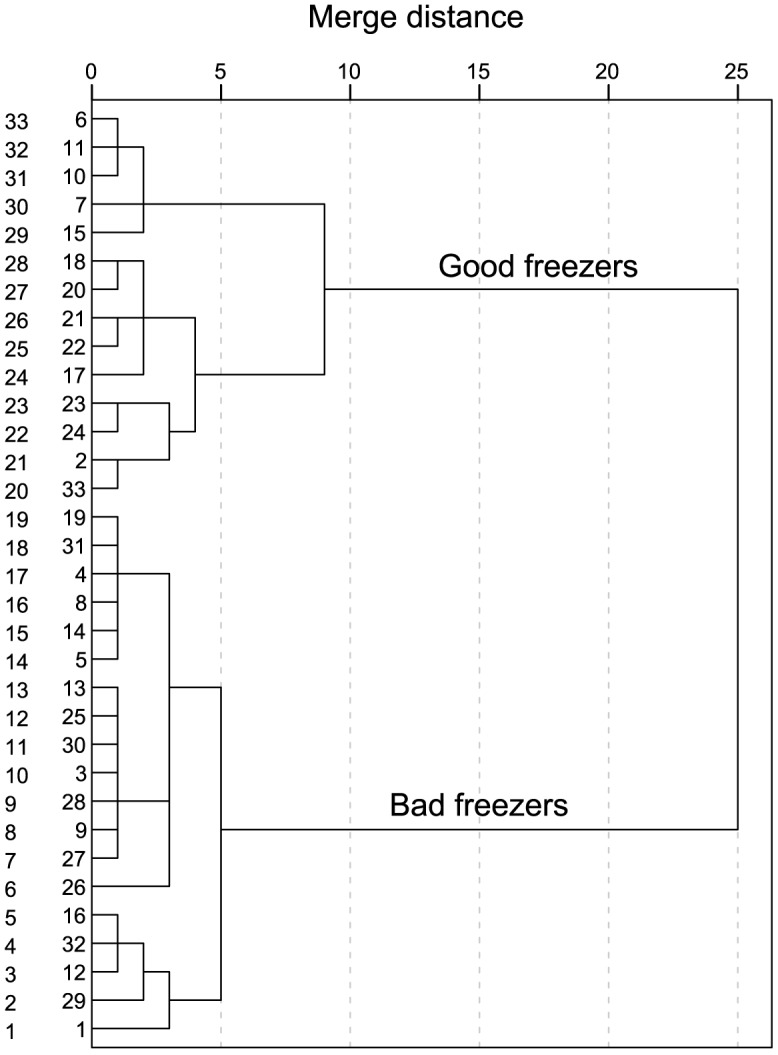
Cluster dendrogram analysis showing red deer sperm freezability. Fourteen males were identified as good freezers, whereas 19 as bad freezers.

The cluster dendrogram analysis of sperm freezability is shown in Figure 2. Fourteen stags were clearly identified as GF, whereas nineteen were BF. Not only did the GF and the BF show clear differences in the three parameters (VAP, VCL, and VSL) used for the cluster analysis, but they also showed differences in the other sperm functionality parameters. Indeed, at 0 hours post-thawing we found significant differences in the following sperm parameters: motile sperm, t(31) = −2.23, p = 0.033; VAP, t(31) = −3.58, p = 0.001; VCL, t(31) = −4.18, p<0.001; VSL, t(31) = −2.66, p = 0.012; ALH, t(31) = −4.30, p<0.001; YOPRO-1-/PI-, t(27.97) = −2.82, p = 0.009; and MT+, t(26.75) = −2.30, p = 0.029 (Table 3). The differences between the two groups of males in sperm quality were more evident across sperm incubation (Table 3). Thus, sperm kinetics, viability, and organelle functionality were significantly different between the GF and the BF at 2 hours post-thawing as follows: motile sperm, t(31) = −3.87, p<0.001; QM, U = 66.00, p = 0.003; SMI, t(31) = −4.62, p<0.001; VAP, t(31) = −9.66, p<0.001; VCL, t(19.24) = −8.28, p<0.001; VSL, t(31) = −8.08, p<0.001; ALH, t(31) = −7.60, p<0.001; YOPRO-1-/PI-, t(28) = −2.67, p = 0.013; MT+, t(31) = −3.43, p = 0.002; and PNA-, t(31) = −2.69, p = 0.011 (Table 3). On the other hand, the quality of motility, SMI, and acrosomal status were not significantly different between the GF and the BF at 0 hours post-thawing (Table 3).

**Table 3:** Sperm kinetics, viability and organelle status in good freezers (GF, n = 14) and bad freezers (BF, n = 19) at 0 and 2 hours post-thaw.

Assessed parameters	0 hours post-thaw		2 hours post-thaw	
	GF	BF	GF	BF
Sperm kinetics				
VAP (µm/s)	68.96±12.40^a**^	54.44±10.83^b**^	50.43±5.38^a***^	32.47±5.21^b***^
VCL (µm/s)	112.40±20.13^a***^	86.65±15.30^b***^	80.37±9.70^a***^	56.45±5.56^b***^
VSL (µm/s)	37.71±7.02^a*^	31.56±6.24^b*^	26.90±2.27^a***^	18.70±3.25^b***^
ALH (µm)	4.34±0.78^a***^	3.36±0.53^b***^	3.08±0.32^a***^	2.40±0.19^b***^
Motile sperm (%)	62.86±15.53^a*^	50.79±15.30^b*^	51.43±9.89^a***^	36.05±12.20^b***^
QM (0–5)	1.89±0.19^a^	1.83±0.33^a^	1.55±0.11^a**^	1.34±0.22^b**^
SMI (%)	50.36±9.14^a^	43.68±9.80^a^	41.25±5.07^a***^	31.45±6.63^b***^
Sperm viability and organelle status				
Viability (YOPRO-1-/PI-, %)	40.10±5.00^a**^	32.65±9.91^b**^	33.50±4.70^a*^	26.91±9.29^b*^
Active mitochondria (MT+, %)	56.72±7.44^a*^	47.01±16.20^b*^	40.06±18.20^a**^	20.22±15.05^b**^
Intact acrosome (PNA-, %)	86.26±6.30^a^	82.26±6.84^a^	75.24±9.87^a*^	65.57±10.45^b*^

Different superscript letters within the same row differ significantly between GF and BF at 0 and 2 hours post-thaw, respectively (*p<0.05; **p<0.01; ***p<0.001). Data are shown as mean ± SD (standard deviation). GF, good freezers; BF, bad freezers; VAP, average path velocity; VCL, curvilinear velocity; VSL, straight linear velocity; ALH, amplitude of lateral head displacement; QM, quality of motility; SMI, sperm motility index.

### Sperm quality before freezing

There were no significant differences between the GF and the BF before freezing in any of the subjective kinetics parameters evaluated. Indeed, the GF and the BF showed similar subjective motility and, therefore, were expected to have the same freezability. Motility parameters in fresh spermatozoa were: motile sperm (83.21±13.24 vs 80.79±10.17, t(31) = −0.60, p = 0.556), QM (2.25±0.51 vs 2.24±0.48, U = 127.50, p = 0.825), and SMI (64.11±10.63 vs 62.76±8.07, t(31) = −0.41, p = 0.682). Data are shown as the mean ± SD for the GF and the BF, respectively.

### Sperm freezability and morphometry of fresh sperm

**Figure 3:**
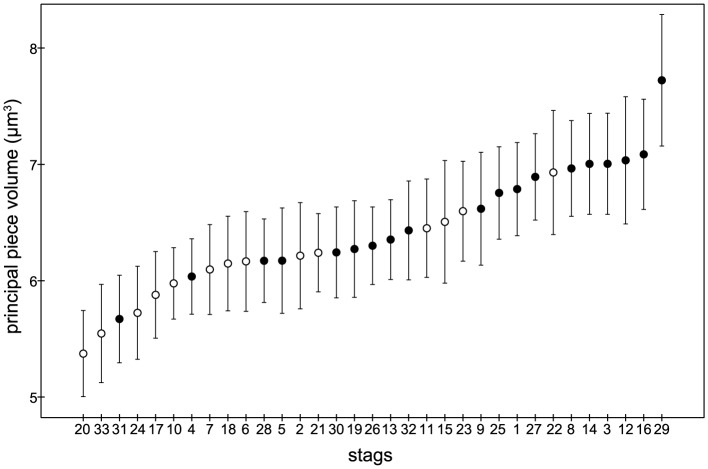
Classification of sperm principal piece volume according to red deer sperm freezability. Good freezers are shown in open circles and bad freezers in closed circles.

The descriptive statistics for the morphometric parameters are shown in Table 4. There were no differences between the GF and the BF in any of the sperm head measurements (Table 4). On the other hand, highly significant differences were observed between groups in regard to sperm flagellum morphometry (Table 4). Thus, the GF exhibited a lower mean principal piece volume than the BF (6.13±0.42 µm^3^ vs. 6.61±0.49 µm^3^, t(31) = 2.93, p = 0.006). Also, the GF exhibited a smaller mean distal midpiece width than the BF (0.73±0.02 µm vs. 0.75±0.03 µm, t(31) = 2.37, p = 0.024). The data are shown as the mean ± SD for the GF and the BF, respectively. Moreover, the flagellum and midpiece volumes together with some sperm lengths (e.g., total sperm and flagellum) showed lower values in the GF than the BF, although the differences were not statistically significant (Table 4). Sperm freezability according to the sperm principal piece volume is shown in Figure 3.

**Table 4:** Overall morphometry of fresh sperm (data derived from 825 spermatozoa from 33 red deer) and differences between good (GF) and bad freezers (BF) in sperm morphometry parameters.

Assessed parameters	Overall (N = 33)			GF (n = 14)	BF (n = 19)	*P*
	Mean ± SD	Range	CV	Mean ± SD	Mean ± SD	
Sperm head						
Width (µm)	5.17±0.12	4.82–5.35	2.35	5.17±0.11	5.16±0.13	0.766
Length (µm)	8.75±0.24	8.35–9.30	2.79	8.77±0.25	8.74±0.25	0.730
Area (µm^2^)	35.51±1.17	33.09–37.94	3.30	35.64±1.30	35.41±1.10	0.597
Perimeter (µm)	22.23±0.43	21.36–23.23	1.92	22.27±0.46	22.20±0.41	0.653
Flagellum and sperm length						
Midpiece width, proximal (µm)	0.94±0.03	0.85–1.03	3.56	0.93±0.05	0.94±0.02	0.382
Midpiece width, distal (µm)	0.74±0.03	0.68–0.80	3.63	0.73±0.02	0.75±0.03	**0.024**
Midpiece length (µm)	12.06±0.26	11.50–12.60	2.12	12.02±0.18	12.08±0.30	0.506
Principal piece length (µm)	41.47±1.26	38.92–44.39	3.04	41.07±1.42	41.76±1.07	0.122
Terminal piece length (µm)	2.68±0.26	2.05–3.21	9.89	2.61±0.28	2.72±0.25	0.248
Flagellum length (µm)	56.20±1.29	53.56–59.05	2.29	55.71±1.41	56.57±1.09	0.057
Midpiece volume (µm^3^)	6.73±0.48	5.68–7.70	7.09	6.57±0.56	6.85±0.38	0.097
Principal piece volume (µm^3^)	6.41±0.51	5.37–7.72	7.98	6.13±0.42	6.61±0.49	**0.006**
Flagellum volume (µm^3^)	12.95±1	10.36–15.22	7.70	12.67±1.24	13.16±0.73	0.161
Sperm length (µm)	64.96±1.29	62.35–67.52	1.99	64.48±1.34	65.31±1.17	0.068

Bold letters show significant differences between GF and BF. SD, standard deviation; CV, coefficient of variation.

### Sperm velocity post-thaw and its relationship with morphometry of fresh sperm

Principal component analysis rendered only one component both at 0 and 2 hours post-thawing. The components explained 91.68% and 83.98% of the variance in sperm velocity at 0 and 2 hours post-thawing, respectively. The PCA are shown in Table 5.

**Table 5:** Results of principal component analysis (PCA) to determine overall sperm velocity at 0 and 2 hours post-thaw.

PCA	0 hours	2 hours
Variables	PC1	PC1
VAP	0.986	0.951
VCL	0.975	0.967
VSL	0.960	0.920
ALH	0.957	0.927
SMI	0.907	0.809
Variance explained (%)	91.676	83.978
Eigenvalue	4.584	4.199
Bartlett's test of sphericity	0.000	0.000
Kaiser-Meyer-Olkin test	0.795	0.744

VAP, average path velocity; VCL, curvilinear velocity; VSL, straight linear velocity; ALH, amplitude of lateral head displacement; SMI, sperm motility index.

**Figure 4:**
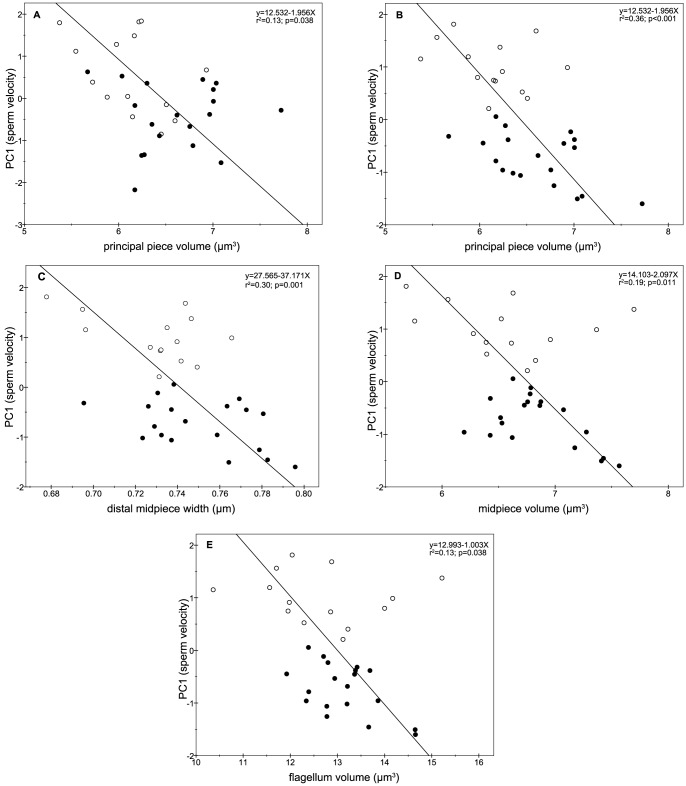
Relationships between sperm velocity and flagellum morphometry. Good freezers are shown in open circles and bad freezers in closed circles: A (0 hours post-thaw); B, C, D, and E (2 hours post-thaw).

None of the morphometric parameters of the sperm head were related with sperm velocity. In contrast, sperm velocity showed strong and negative relationships with sperm flagellum volumes, particularly with the sperm principal piece volume. Indeed, at 0 hours post-thawing, the principal piece volume was the only parameter showing a significant relationship with sperm velocity (r = −0.36; p = 0.038) (Figure 4A). In addition, such a relationship reached the highest values at 2 hours post-thawing (r = −0.60; p<0.001) (Figure 4B). On the other hand, sperm velocity was also negatively correlated with distal midpiece width (r = −0.55; p = 0.001) (Figure 4C) and with midpiece and flagellum volumes (r = −0.44; p = 0.011 and r = −0.36; p = 0.038) at 2 hours post-thawing (Figures 4D–E, respectively).

## Discussion

In this study, for the first time, the volumes of the flagellum structures in fresh sperm have been determined to predict sperm freezability in red deer. Our results clearly show that sperm with a higher principal piece volume freeze worse, that is, the GF have a lower principal piece volume than the BF. We also found that sperm velocity is strongly and negatively related with the volumes of the flagellum structures. Sperm velocity is crucial in the process of fertilization in a large number of taxa (including fish [38], birds [39], and mammals [34], [40]). Moreover, VAP, VCL, and VSL have been proven to be good indicators of sperm freezability in red deer as previously described in canine sperm [35], and in turn, these parameters are closely related with fertility in red deer using thawed sperm [34].

The evaluation of sperm function throughout post-thawing and sperm incubation provides additional information about the quality of the spermatozoa [41], [42] and is more closely related to sperm fertility than those sperm assessed immediately after thawing [43], [44]. According to this assumption, we found more and stronger relationships between sperm velocity and the volumes of the flagellum structures at 2 hours than at 0 hours post-thawing. For example, we did not find any differences between the GF and the BF in acrosomal status at 0 hours post-thawing, but we did at 2 hours post-thawing. This is probably because if the sperm membrane or other structures (e.g. axoneme) were disrupted, this damage was not manifested immediately upon thawing, but occurred during post-thaw re-warming within specific temperatures [23]. Thus, the damage in this structure would be higher due to thermal stress during sperm incubation, inasmuch as sperm thawing is more deleterious than sperm freezing [23], [45] and can result in more morphological damage [46].

In the present work, we did not find any significant differences between the GF and the BF in sperm head size, and also none of the morphometric parameters of this structure showed any relationship with sperm velocity. By contrast, Esteso et al. [14] found that sperm head size is related with sperm freezability in red deer (i.e., increased head size entails a poor sperm freezability). However, Esteso et al. [14] classified sperm donors as a GF or BF using the sperm motility index, instead of a CASA system, together with acrosomal status and membrane stability. Within this context, we found that the intact acrosome is negatively related with sperm head size at 0 hours post-thawing (Figure S1). On the other hand, the sperm principal piece volume and distal midpiece width showed significant differences between the GF and the BF, and they are also negatively correlated with sperm velocity. According to this fact, Peña et al. [3] found that midpiece width is a predictor of post-thawing boar sperm motility. In our study, the principal piece volume is mainly determined by the distal midpiece width (Figure S2), which can explain their similar relationships with sperm velocity, and also the differences found between the GF and the BF in these sperm measures. The damage caused by sperm freezing protocols to the sperm head and tail membranes may occur independently: an intact tail membrane does not necessarily indicate an intact sperm head membrane and vice versa [47]. For example, after the freezing-thawing process, the disruption of the sperm head membrane occurs more easily than the tail in human and ram sperm [23], [47], whereas the flagellum membrane is more vulnerable to the cryopreservation process in equine sperm [7]. Sperm with an intact head membrane but a damaged flagellum are most likely immotile, explaining the low fertilization rates with frozen/thawed sperm [7], [48]; therefore, these cells should be included in the dead rather than the alive category [48]. In our study, the sperm flagellum is likely more sensitive to the freezing/thawing process than the sperm head or, at least, a damaged flagellum negatively affects sperm kinetics more than a damaged sperm head.

The fragility of the sperm flagellum and its ability to withstand the freezing/thawing process has been reported in many studies. The addition of glycerol as a cryoprotectant agent alters sperm functionality, mainly during the post-thawing sperm incubation stage [49], showing an increase in the proportion of epididymal spermatozoa with axonemal vacuoles, damaged plasma membranes, and abnormal mitochondria when compared to media without glycerol [50]. Furthermore, the cytoskeleton is responsible for the appropriate cell volume regulation and its stability is highly altered by the cryopreservation process, causing its proteins (e.g., F-actin) to become more fragile [13], [24], [51], [52]. Thus, in red deer, a higher volume of the sperm flagellum might result in an increased amount of ice crystals and increased formation of axonemal vacuoles during the freezing/thawing process, adversely affecting flagellum integrity and consequently, cell volume regulation and sperm velocity. Supporting this hypothesis, Correa et al. [53] suggested a direct connection between cell volume regulation, flagellum morphology, motility, and the actin cytoskeleton in the sublethal damage that occurs during osmotic stress and, potentially, during cryopreservation. On the other hand, we did not find any significant differences between the GF and the BF in the midpiece or flagellum size which contain sperm mitochondria and the whole sperm axoneme, respectively. On the contrary, the sperm principal piece volume differs between the GF and the BF. Such a result might be related to the fibrous sheath, which is located along the principal piece and linked with sperm kinetics [54]. Thus, differences among males in the principal piece volume could differently affect their sperm freezability. Furthermore, it is thought that the fibrous sheath plays a mechanical role in sperm motility, providing a rigid support to the flagellum and determining its planar beat [55], [56]. Additionally, glycolysis is carried out along the length of the principal piece and this, instead of oxidative phosphorylation in the midpiece, is the most important source of ATP for the tail [57]. At least one fibrous sheath protein may act to protect sperm from oxidative stress, which could interfere with sperm motility or cause DNA damage [58]. Therefore, the proteome and the general structure of the fibrous sheath could be potentially damaged by the sperm freezing and thawing process, particularly in those sperm with a higher sperm principal piece volume, causing a decrease in sperm function mainly during sperm incubation.

In conclusion, our results provide evidence that the volumes of the flagellum structures are a determinant to predict post-thaw sperm velocity in red deer, and the BF have a higher sperm principal piece volume than the GF. In contrast, sperm head size is not a good predictor of post-thaw sperm velocity in red deer spermatozoa. However, further studies, including additional analyses such as freeze-fracture electron microscopy observations of spermatozoa [50], [59] and the evaluation of sperm tail membrane integrity by light microscopy [48] during sperm cooling and especially during sperm warming, are necessary in order to confirm our findings. On the other hand, our subsequent studies will be directed towards the use of electron microscopy, to assess sperm flagellum morphometry in more depth, and also to measure their internal structures (axoneme, fibrous sheath, etc.). Our results clearly show that a higher principal piece volume results in poor sperm freezability, and highlights the key role of the volume of flagellum structures in sperm cryopreservation success.

## Supporting Information

Figure S1 **Relationships between sperm head perimeter and intact acrosome at 0 hours of sperm thawing (r = −0.365; p = 0.037).**
(JPG)

Figure S2 **Relationships between sperm principal piece volume and distal midpiece width (r = 0.93; p<0.0001).**
(JPG)

Table S1 **Individual mean morphometry and subjective motility of fresh red deer spermatozoa (N = 33).**
(DOC)

Table S2 **Individual mean kinetics, viability, and organelle status of red deer spermatozoa at 0 hours post-thaw (N = 33).**
(DOC)

Table S3 **Individual mean kinetics, viability, and organelle status of red deer spermatozoa at 2 hours post-thaw (N = 33).**
(DOC)
