# Supplementary figures and images for: Sperm Flagellum Volume Determines Freezability in Red Deer Spermatozoa
Source: PLoS One. 2014 Nov 7;9(11):e112382. doi: 10.1371/journal.pone.0112382 (PMC4224448; doi:10.1371/journal.pone.0112382)

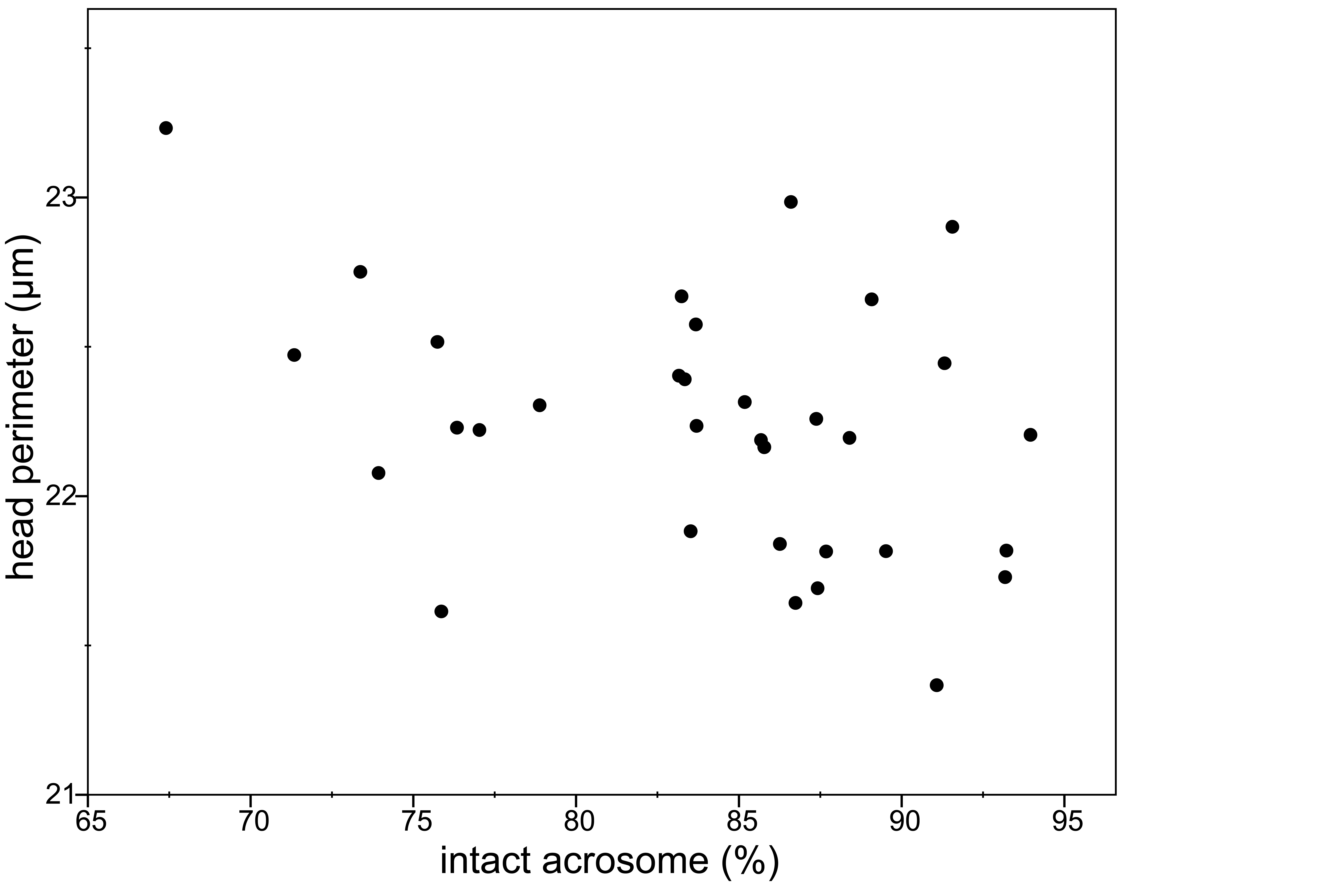

Supplement: Figure S1 — Relationships between sperm head perimeter and intact acrosome at 0 hours of sperm thawing (r = −0.365; p = 0.037). (JPG) [file pone.0112382.s001.jpg]

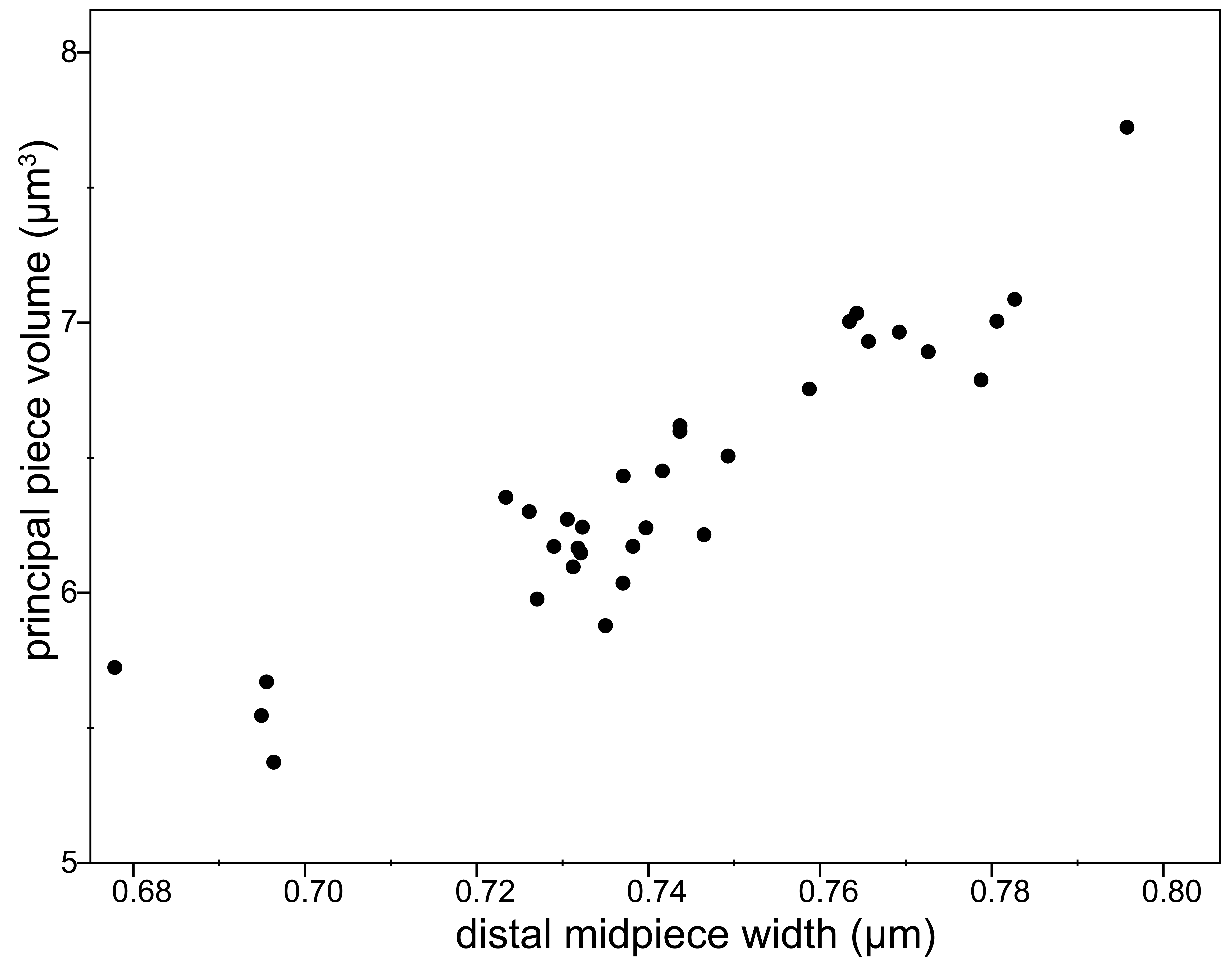

Supplement: Figure S2 — Relationships between sperm principal piece volume and distal midpiece width (r = 0.93; p<0.0001). (JPG) [file pone.0112382.s002.jpg]
