# Supplementary material for: Sperm Flagellum Volume Determines Freezability in Red Deer Spermatozoa
Source: PLoS One. 2014 Nov 7;9(11):e112382. doi: 10.1371/journal.pone.0112382 (PMC4224448; doi:10.1371/journal.pone.0112382)
Supplement: Table S1 — Individual mean morphometry and subjective motility of fresh red deer spermatozoa (N = 33). (DOC) [file pone.0112382.s003.doc]

| **MALE** | **HW** | **HL** | **PMW** | **DMW** | **ML** | **FL** | **TPL** | **MS** | **QM** |
| --- | --- | --- | --- | --- | --- | --- | --- | --- | --- |
| 1 | 5.12 | 8.79 | 0.94 | 0.78 | 12.25 | 54.78 | 2.76 | 80 | 2 |
| 2 | 5.29 | 8.90 | 1.03 | 0.75 | 12.28 | 54.53 | 3.14 | 80 | 2.5 |
| 3 | 5.16 | 8.42 | 0.94 | 0.78 | 12.10 | 55.77 | 2.82 | 70 | 2.5 |
| 4 | 5.06 | 8.55 | 0.91 | 0.74 | 12.54 | 54.80 | 2.25 | 65 | 1.5 |
| 5 | 5.28 | 8.79 | 0.93 | 0.74 | 12.08 | 54.99 | 2.47 | 65 | 1.5 |
| 6 | 5.06 | 8.82 | 0.94 | 0.73 | 11.97 | 55.65 | 2.42 | 75 | 1.5 |
| 7 | 5.25 | 8.45 | 0.95 | 0.73 | 12.06 | 55.34 | 2.25 | 65 | 1.5 |
| 8 | 5.35 | 8.61 | 0.93 | 0.77 | 11.92 | 56.65 | 2.78 | 90 | 2 |
| 9 | 4.82 | 9.20 | 0.94 | 0.74 | 11.62 | 56.94 | 3.10 | 90 | 2.5 |
| 10 | 5.23 | 9.09 | 0.98 | 0.73 | 11.97 | 55.00 | 2.47 | 70 | 2.5 |
| 11 | 5.21 | 8.63 | 0.91 | 0.74 | 11.82 | 56.34 | 2.72 | 75 | 2.5 |
| 12 | 5.09 | 9.06 | 0.98 | 0.76 | 12.26 | 57.87 | 2.79 | 80 | 2.5 |
| 13 | 5.18 | 8.88 | 0.93 | 0.72 | 11.87 | 58.06 | 2.52 | 80 | 2 |
| 14 | 5.09 | 8.46 | 0.94 | 0.76 | 11.96 | 57.61 | 2.75 | 80 | 2.5 |
| 15 | 5.28 | 8.97 | 0.95 | 0.75 | 11.93 | 55.77 | 2.86 | 85 | 2.5 |
| 16 | 5.35 | 8.59 | 0.96 | 0.78 | 12.34 | 56.23 | 2.94 | 85 | 2 |
| 17 | 5.25 | 8.79 | 0.91 | 0.74 | 12.24 | 53.56 | 2.40 | 100 | 2.5 |
| 18 | 5.10 | 8.90 | 0.91 | 0.73 | 12.06 | 55.56 | 2.60 | 100 | 2.5 |
| 19 | 5.01 | 8.64 | 0.92 | 0.73 | 12.59 | 57.20 | 2.89 | 100 | 2.5 |
| 20 | 4.89 | 9.01 | 0.85 | 0.70 | 12.11 | 54.12 | 2.63 | 95 | 2.5 |
| 21 | 5.19 | 8.75 | 0.91 | 0.74 | 11.67 | 55.02 | 2.64 | 100 | 2.5 |
| 22 | 5.25 | 9.12 | 0.97 | 0.77 | 12.31 | 57.08 | 2.72 | 60 | 1.5 |
| 23 | 5.16 | 8.53 | 0.92 | 0.74 | 12.03 | 57.32 | 2.80 | 80 | 1.5 |
| 24 | 5.22 | 8.48 | 0.88 | 0.68 | 11.78 | 59.05 | 2.88 | 90 | 2.5 |
| 25 | 5.20 | 9.30 | 0.96 | 0.76 | 12.42 | 56.98 | 2.55 | 90 | 2.5 |
| 26 | 5.22 | 8.75 | 0.95 | 0.73 | 12.13 | 57.60 | 2.93 | 80 | 3 |
| 27 | 5.20 | 8.53 | 0.96 | 0.77 | 11.60 | 55.50 | 2.39 | 95 | 2.5 |
| 28 | 5.15 | 8.77 | 0.92 | 0.73 | 12.22 | 56.35 | 2.58 | 65 | 2.5 |
| 29 | 5.24 | 8.68 | 0.98 | 0.80 | 12.15 | 58.30 | 2.61 | 75 | 3 |
| 30 | 5.10 | 8.97 | 0.92 | 0.73 | 11.50 | 55.71 | 2.44 | 80 | 1.5 |
| 31 | 5.08 | 8.51 | 0.95 | 0.70 | 11.93 | 56.40 | 2.90 | 75 | 1.5 |
| 32 | 5.35 | 8.57 | 0.92 | 0.74 | 12.13 | 57.05 | 3.21 | 90 | 2.5 |
| 33 | 5.04 | 8.35 | 0.90 | 0.69 | 12.08 | 55.56 | 2.05 | 90 | 3 |

**TABLE S1.** Individual mean morphometry and subjective motility of fresh red deer spermatozoa (N=33).

HW (head width, μm), HL (head length, μm), PMW (proximal midpiece width, μm), DMW (distal midpiece width, μm), ML (midpiece length, μm), FL (flagellum length, μm), TPL (terminal piece length, μm), MS (motile sperm, %), and QM (quality of motility, 0-5).
