# Supplementary material for: Sperm Flagellum Volume Determines Freezability in Red Deer Spermatozoa
Source: PLoS One. 2014 Nov 7;9(11):e112382. doi: 10.1371/journal.pone.0112382 (PMC4224448; doi:10.1371/journal.pone.0112382)
Supplement: Table S2 — Individual mean kinetics, viability, and organelle status of red deer spermatozoa at 0 hours post-thaw (N = 33). (DOC) [file pone.0112382.s004.doc]

| **MALE** | **MS** | **QM** | **VAP** | **VCL** | **VSL** | **ALH** | **YP-/PI-** | **MT+** | **PNA-** |
| --- | --- | --- | --- | --- | --- | --- | --- | --- | --- |
| 1 | 30 | 1.75 | 46.25 | 77.32 | 26.44 | 3.04 | 29.60 | 37.98 | 76.34 |
| 2 | 75 | 1.75 | 85.75 | 144.36 | 46.57 | 5.40 | 46.08 | 65.66 | 89.08 |
| 3 | 60 | 1.75 | 59.73 | 97.88 | 32.28 | 3.68 | 37.44 | 53.44 | 86.74 |
| 4 | 65 | 2.00 | 68.11 | 106.83 | 38.46 | 4.05 | 36.46 | 38.06 | 93.18 |
| 5 | 55 | 2.00 | 55.81 | 89.45 | 34.97 | 3.56 | 33.16 | 45.40 | 91.32 |
| 6 | 75 | 2.00 | 82.41 | 129.92 | 43.83 | 5.00 | 44.36 | 62.22 | 93.96 |
| 7 | 65 | 1.75 | 61.29 | 94.78 | 35.72 | 3.57 | 35.56 | 45.42 | 93.22 |
| 8 | 60 | 2.00 | 56.95 | 82.68 | 32.17 | 3.06 | 47.18 | 65.64 | 77.03 |
| 9 | 50 | 2.00 | 53.27 | 89.69 | 29.70 | 3.61 | 45.36 | 78.51 | 83.68 |
| 10 | 80 | 2.00 | 74.04 | 119.77 | 44.38 | 4.86 | 44.50 | 62.62 | 91.56 |
| 11 | 40 | 1.75 | 50.29 | 83.20 | 27.03 | 3.18 | 39.24 | 52.06 | 73.93 |
| 12 | 60 | 1.75 | 63.88 | 110.33 | 35.41 | 4.26 | 23.56 | 31.54 | 83.24 |
| 13 | 35 | 2.00 | 55.04 | 85.70 | 31.92 | 3.13 | 19.26 | 28.02 | 71.34 |
| 14 | 60 | 2.00 | 61.03 | 97.96 | 38.90 | 3.74 | 33.06 | 53.32 | 75.86 |
| 15 | 55 | 2.00 | 61.81 | 89.09 | 35.01 | 3.27 | 37.36 | 53.39 | 73.37 |
| 16 | 30 | 1.00 | 41.02 | 68.46 | 24.75 | 2.90 | 41.62 | 64.04 | 85.68 |
| 17 | 55 | 1.75 | 61.88 | 103.85 | 29.89 | 4.16 | 42.10 | 57.44 | 83.16 |
| 18 | 35 | 1.75 | 59.61 | 91.92 | 31.25 | 3.57 | 28.54 | 42.48 | 83.34 |
| 19 | 35 | 1.50 | 43.11 | 68.88 | 25.82 | 2.80 | 27.20 | 39.14 | 87.68 |
| 20 | 75 | 2.25 | 87.21 | 132.28 | 49.03 | 5.03 | 44.42 | 65.98 | 85.18 |
| 21 | 85 | 2.25 | 82.53 | 136.89 | 44.13 | 5.47 | 42.66 | 59.82 | 87.38 |
| 22 | 60 | 1.75 | 70.40 | 116.94 | 38.39 | 4.48 | 38.14 | 59.68 | 86.60 |
| 23 | 45 | 1.75 | 50.95 | 90.88 | 28.94 | 3.66 | 34.22 | 47.50 | 86.26 |
| 24 | 60 | 1.75 | 62.68 | 113.05 | 35.62 | 4.31 | 39.40 | 58.78 | 89.52 |
| 25 | 40 | 2.00 | 52.84 | 83.11 | 30.23 | 3.17 | 31.20 | 48.26 | 67.40 |
| 26 | 70 | 2.00 | 66.16 | 100.72 | 35.14 | 3.93 | 43.10 | 64.49 | 78.88 |
| 27 | 75 | 2.25 | 64.91 | 95.50 | 39.02 | 3.74 | 51.16 | 70.01 | 83.52 |
| 28 | 40 | 1.00 | 26.08 | 55.49 | 15.56 | 2.49 | 26.32 | 34.80 | 83.70 |
| 29 | 65 | 2.00 | 55.73 | 83.88 | 33.31 | 3.14 | 34.44 | 56.94 | 88.40 |
| 30 | 25 | 2.00 | 43.67 | 65.99 | 27.86 | 2.57 | 18.46 | 26.42 | 75.74 |
| 31 | 65 | 2.00 | 68.68 | 108.11 | 41.21 | 4.05 | 20.22 | 28.68 | 87.42 |
| 32 | 45 | 1.75 | 52.11 | 78.48 | 26.46 | 2.96 | 21.62 | 28.42 | 85.78 |
| 33 | 75 | 2.00 | 74.60 | 126.69 | 38.13 | 4.83 | 44.72 | 60.98 | 91.08 |

**TABLE S2**. Individual mean kinetics, viability, and organelle status of red deer spermatozoa at 0 hours post-thaw (N=33).

MS (motile sperm, %), QM (quality of motility, 0-5), VAP (average path velocity, μm/s), VCL (curvilinear velocity, μm/s), VSL (straight linear velocity, μm/s), ALH (amplitude of lateral head displacement, μm), YP-/PI- (viability, %), MT+ (active mitochondria, %) and, PNA- (intact acrosome, %).
