# Supplementary material for: Sperm Flagellum Volume Determines Freezability in Red Deer Spermatozoa
Source: PLoS One. 2014 Nov 7;9(11):e112382. doi: 10.1371/journal.pone.0112382 (PMC4224448; doi:10.1371/journal.pone.0112382)
Supplement: Table S3 — Individual mean kinetics, viability, and organelle status of red deer spermatozoa at 2 hours post-thaw (N = 33). (DOC) [file pone.0112382.s005.doc]

| **MALE** | **MS** | **QM** | **VAP** | **VCL** | **VSL** | **ALH** | **YP-/PI-** | **MT+** | **PNA-** |
| --- | --- | --- | --- | --- | --- | --- | --- | --- | --- |
| 1 | 30 | 1.00 | 23.19 | 57.04 | 14.57 | 2.53 | 25.56 | 30.54 | 64.76 |
| 2 | 55 | 1.50 | 50.16 | 87.23 | 29.15 | 3.37 | 36.94 | 46.46 | 80.82 |
| 3 | 40 | 1.50 | 31.86 | 60.72 | 17.56 | 2.58 | 29.30 | 39.08 | 72.24 |
| 4 | 35 | 1.25 | 36.43 | 61.47 | 21.16 | 2.53 | 23.50 | 25.94 | 81.32 |
| 5 | 50 | 1.50 | 38.22 | 64.16 | 22.31 | 2.73 | 31.14 | 39.12 | 84.80 |
| 6 | 50 | 1.75 | 46.37 | 72.86 | 25.63 | 2.93 | 37.38 | 53.44 | 90.72 |
| 7 | 45 | 1.50 | 39.64 | 70.23 | 22.85 | 2.83 | 27.06 | 37.50 | 82.80 |
| 8 | 50 | 1.50 | 37.40 | 59.82 | 20.34 | 2.51 | 38.40 | 1.25 | 57.55 |
| 9 | 45 | 1.50 | 30.97 | 57.60 | 16.01 | 2.45 | 38.62 | 3.12 | 55.46 |
| 10 | 65 | 1.50 | 44.66 | 71.35 | 25.87 | 3.06 | 35.76 | 50.02 | 87.20 |
| 11 | 45 | 1.50 | 45.69 | 72.01 | 25.50 | 2.91 | 29.38 | 0.88 | 54.19 |
| 12 | 25 | 1.00 | 26.42 | 51.14 | 14.12 | 2.24 | 18.66 | 23.48 | 76.80 |
| 13 | 25 | 1.50 | 30.27 | 53.63 | 17.37 | 2.25 | 13.20 | 2.00 | 54.69 |
| 14 | 40 | 1.50 | 36.76 | 57.64 | 21.81 | 2.40 | 30.28 | 4.47 | 62.43 |
| 15 | 50 | 1.50 | 47.05 | 64.34 | 29.44 | 2.42 | 31.88 | 1.06 | 61.03 |
| 16 | 25 | 1.00 | 28.83 | 48.29 | 15.61 | 2.20 | 37.78 | 48.84 | 75.36 |
| 17 | 40 | 1.50 | 57.16 | 85.93 | 28.22 | 3.15 | 35.18 | 50.88 | 71.62 |
| 18 | 35 | 1.75 | 53.35 | 76.39 | 27.83 | 2.68 | 23.94 | 27.32 | 67.86 |
| 19 | 35 | 1.50 | 42.26 | 62.11 | 24.09 | 2.45 | 20.36 | 27.36 | 71.58 |
| 20 | 50 | 1.50 | 56.30 | 79.53 | 29.15 | 3.02 | 38.00 | 56.08 | 82.22 |
| 21 | 55 | 1.50 | 53.66 | 79.99 | 23.33 | 3.05 | 39.50 | 50.82 | 75.42 |
| 22 | 45 | 1.50 | 52.04 | 84.76 | 24.71 | 3.20 | 33.50 | 49.82 | 78.52 |
| 23 | 50 | 1.50 | 56.65 | 95.40 | 29.36 | 3.52 | 28.10 | 38.18 | 70.58 |
| 24 | 65 | 1.50 | 55.72 | 95.45 | 28.68 | 3.55 | 36.48 | 47.16 | 75.68 |
| 25 | 25 | 1.50 | 29.52 | 53.57 | 18.20 | 2.34 | 26.28 | 6.50 | 55.25 |
| 26 | 60 | 1.00 | 30.24 | 65.08 | 18.14 | 2.75 | 41.32 | 2.34 | 59.29 |
| 27 | 55 | 1.50 | 36.78 | 54.92 | 19.68 | 2.22 | 36.42 | 11.83 | 53.77 |
| 28 | 25 | 1.25 | 32.34 | 58.28 | 18.96 | 2.54 | 22.64 | 25.18 | 73.96 |
| 29 | 40 | 1.00 | 24.21 | 43.92 | 14.40 | 2.05 | 33.84 | 41.74 | 75.62 |
| 30 | 15 | 1.50 | 32.01 | 54.48 | 20.09 | 2.24 | 13.26 | 13.68 | 57.46 |
| 31 | 25 | 1.50 | 39.48 | 59.12 | 25.09 | 2.39 | 15.14 | 18.50 | 62.84 |
| 32 | 40 | 1.50 | 29.67 | 49.53 | 15.69 | 2.16 | 15.50 | 19.20 | 50.74 |
| 33 | 70 | 1.75 | 47.62 | 89.78 | 26.76 | 3.44 | 35.90 | 51.18 | 74.66 |

**TABLE S3**. Individual mean kinetics, viability, and organelle status of red deer spermatozoa at 2 hours post-thaw (N=33).

MS (motile sperm, %), QM (quality of motility, 0-5), VAP (average path velocity, μm/s), VCL (curvilinear velocity, μm/s), VSL (straight linear velocity, μm/s), ALH (amplitude of lateral head displacement, μm), YP-/PI- (viability, %), MT+ (Active mitochondria, %) and, PNA- (intact acrosome, %).
